# Pressure injury prevention in older people: construction and validation of an instrument for caregivers

**DOI:** 10.1590/0034-7167-2021-0930

**Published:** 2023-01-30

**Authors:** Suellen Duarte de Oliveira Matos, Ana Paula Marques de Andrade Souza, Margarida da Silva Neves de Abreu, Anne Carolinne Marie dos Santos Gomes, Jacira dos Santos Oliveira, Mirian Alves da Silva, Maria Júlia Guimarães Oliveira Soares, Simone Helena dos Santos Oliveira

**Affiliations:** I Faculdade de Enfermagem Nova Esperança. João Pessoa, Paraíba, Brazil; II Universidade Federal da Paraíba. João Pessoa, Paraíba, Brazil; III Escola Superior de Enfermagem do Porto. Porto, Portugal

**Keywords:** Validation Study, Pressure Ulcer, Caregivers, Health Knowledge, Attitudes, Practice, Nursing Methodology Research., Estudio de Validación, Úlcera por Presión, Cuidadores, Conocimientos, Actitudes y Práctica en Salud, Investigación Metodológica en Enfermería., Estudo de Validação, Lesão por Pressão, Cuidadores, Conhecimentos, Atitudes e Prática em Saúde, Pesquisa Metodológica em Enfermagem.

## Abstract

**Objectives:**

to construct and validate an instrument to assess the knowledge, attitudes, and practices related to pressure injury prevention among caregivers of institutionalized older people.

**Methods:**

this is a three-stage methodological study that consisted of instrument construction, analysis by experts, and semantic and appearance analysis, with 78 participants, observing the validation process steps for psychometric instruments in the criteria of clarity and relevance.

**Results:**

in the Delphi I round, the validity index of the general content in the clarity criterion was 0.66, in relevance 0.85, and the Kappa value was >0.76. In Delphi II, clarity was 0.95, relevance 1.00, and the Kappa value was >0.97.

**Conclusions:**

this is a valid instrument in terms of content and appearance, which allows further analysis of its reliability for the measurement of the constructs for which it is intended. Therefore, it can be considered a tool for care management in pressure injury prevention.

## RESUMO

**Objetivos:**

construir e validar instrumento relacionado à prevenção de lesão por pressão para avaliação do conhecimento, da atitude e da prática de cuidadores de idosos institucionalizados.

**Métodos:**

estudo metodológico realizado em três fases: construção do instrumento, análise dos juízes e análise semântica e aparente, com 78 participantes, seguindo as etapas do processo de validação de instrumentos psicométricos para os critérios clareza e pertinência.

**Resultados:**

na rodada *Delphi* I, o índice de validade de conteúdo global do critério “clareza” foi de 0,66, “pertinência” 0,85 e valor de *Kappa* > 0,76. Em *Delphi* II, o critério de “clareza” foi de 0,95, “pertinência” 1,00 e valor de *Kappa* > 0,97.

**Conclusões:**

dispõe-se de instrumento válido quanto a conteúdo e aparência para dar seguimento à análise da sua confiabilidade em medir os construtos aos quais se propõe, para que possa servir de ferramenta para gestão do cuidado na prevenção de lesão por pressão.

## RESUMEN

**Objetivos:**

construir y validar un instrumento pera evaluación del conocimiento, actitud y práctica relacionado a la prevención de lesiones por presión entre cuidadores de ancianos institucionalizados.

**Métodos:**

estudio metodológico realizado en tres fases: construcción del instrumento, análisis de los evaluadores y análisis semántico y de apariencia, con 78 participantes siguiendo las etapas del proceso de validación de instrumentos psicométricos para los criterios claridad y pertinencia.

**Resultados:**

en la ronda Delphi I el índice de validez de contenido general del criterio “claridad” fue de 0,66, “pertinencia” 0,85, y valor de Kappa > 0,76. En Delphi II, el criterio de “claridad” fue 0,95, “pertinencia” 1,00 y valor de Kappa > 0,97.

**Conclusiones:**

se tiene un instrumento válido respecto de contenidos y apariencia, para continuar analizando su confiabilidad para medir los constructos a los cuales se propone, para que sirva como herramienta de gestión del cuidado en la prevención de lesiones por presión.

## INTRODUCTION

Older people are at higher risk for developing pressure injuries (PIs) due to conditions related to the aging process and clinical conditions that involve reduced mobility. Pressure injuries are a serious public health problem that can generate physical and emotional disorders, affecting morbidity and mortality^(1)^.

Due to PI impact on health, strategic planning must be adopted to identify good care practices for caregivers of long-term care facilities (LTCFs) in order to incorporate effective procedures for PI prevention. With this objective, nurses who work in these scenarios assume the role of care planning and supervision, and provide guidance to caregivers regarding daily care to maintain skin integrity, according to planned activities.

For caregivers, the importance of knowledge, attitudes, and practices (KAP) is emphasized in the adoption of PI-related preventive measures, since they help perform tasks like personal care, feeding, repositioning, skin hydration, and other inherent care activities^(2)^.

The KAP survey, designed to collect data about what a specific population knows, thinks, and practices in relation to a problem^(3)^ to favor the identification of more effective interventions, was used as a guide for the development of the assessment instrument. Its structure was based on the preventive measures described in the Pressure Injury Prevention Protocol issued by the Ministry of Health^(4)^, the guidelines of the National Pressure Injury Advisory Panel, and GVIMS/GGTES Technical Note nº 03/2017^(5-6)^.

Studies using the KAP survey are widely conducted nationwide and worldwide, with some of them focused on caregivers of older people^(7-8)^. Although studies have used the KAP survey for caregivers of older people, PI prevention has not been addressed using this instrument so far. Therefore, it is crucial to assess the knowledge, attitudes, and practices of caregivers in the prevention of pressure injuries in older people living in long-term care facilities.

The construction of an instrument for this population can provide relevant information about this issue and support the development of more effective and feasible intervention strategies to reduce PI rates among this group of patients.

## OBJECTIVES

To construct and validate an instrument to assess the knowledge, attitudes, and practices related to pressure injury prevention among caregivers of institutionalized older people.

## METHODS

### Ethical aspects

This study was approved by the Research Ethics Committee of the Health Science Center, at the Federal University of Paraíba (UFPB), respecting the ethical aspects of research involving human beings issued in Resolution 466/12 of the National Health Council^(9)^.

### Study design, period and setting

This is a three-stage methodological study, including instrument construction, analysis by experts, and semantic and appearance analysis, according to the validation process stages defined by Pasquali^(10)^ for psychometric instruments, as regards theoretical procedures. Data collection was conducted between August 2018 and September 2019 via electronic means and in person in the Technical Health School and the Clinical Nursing Department of the Federal University of Paraíba (UFPB).

### Population and sample

A total of 78 participants took part in the study, respecting the validation process stages defined by Pasquali^(10)^ for psychometric instruments: theoretical pole using a theoretical analysis for content validity, according to the criteria of clarity and relevance, and semantic analysis to confirm whether the items can be understood by the target population; empirical pole, with sample definition, development of steps and techniques for pilot instrument application and valid data collection to check the psychometric quality of the instrument; and analytical pole, using the item content validity index (I-CVI), the scale content validity index (S-CVI), and Cohen’s kappa coefficient.

Experts were selected via electronic search, based on topics such as older people, caregivers of institutionalized older people, pressure injury, and KAP survey. Their résumés were assessed via Lattes platform of the National Council for Scientific and Technological Development (CNPq). A total of 13 experts were invited and 12 of them agreed to participate in this study; 11 answered the evaluation form in the first round and only 10 in the second round, with the latter constituting the sample in this stage, meeting the criteria defined by Pasquali^(10)^.

In addition, the selection criteria were adapted from those used by Fehring^(11)^ and Fehring^(12)^: health professionals with at least a master’s degree (4 points); who have written a dissertation on the topic of interest regarding older people (1 point); who published articles about the subject in a leading journal in the field, as the main author (2 points); who published articles about the subject in a leading journal in the field, as the secondary author (2 points); with a doctor’s degree in nursing (2 points); specialization in geriatric health or public health and wounds (2 points); and clinical experience of at least one year with older people, caregivers of institutionalized older people, pressure injuries, and KAP surveys (1 point). The criterion considered in the model was the attribution of a minimum and maximum score between 5 and 14 points, so an expert was considered adequate to participate in the assessment. The sum of points obtained from the selected experts showed a minimum and maximum score of 11 and 14, respectively.

The experts received a formal invitation through email, together with an informed consent form, a sociodemographic questionnaire, and a link to an online form, containing a summary of the concept of knowledge, attitudes, and practices (KAP), and the definitions for the criteria of clarity and relevance, assessed in a 4-point Likert scale with the attributes: 1 = not clear or not relevant, 2 = little clear or little relevant, 3 = clear or relevant, and 4 = very clear or very relevant.

After content validation, still in the theoretical procedure, a semantic analysis was performed to assess the instrument clarity. Students in the conclusion phase of a technical course for caregivers of older people, linked with the Health Technical School, were invited to participate in our study, selected from a simple stratified sampling plan. Of all 45 students, 27 were in class 1 and 18 in class 2. When proposing the theoretical analysis of the instrument, after semantic validation, to technical course students who are similar to the target population, it is assumed that if the items of the instrument are understandable to this group, it will certainly be to caregivers of older people.

As part of the semantic analysis, an appearance analysis was conducted to test the instrument with a group whose educational level was higher than that of the target population. For this sample, 23 higher education professors were selected, and individually invited to participate in the study.

### Study protocol

The instrument development was based on the KAP survey, which is a formative assessment that collects data from a specific population to measure, through a number of questions^(3)^, what they know, think, and how they act in relation to a given problem.

The instrument was called the Knowledge, Attitudes, and Practices Survey with Caregivers of Older People on Pressure Injury Prevention (*Inquérito conhecimento, atitude e prática de cuidadores de idosos sobre prevenção de lesão por pressão* - InqCAP-CIPLP) and its first version consisted of 19 questions, divided into three sections: 1) knowledge (questions 01 to 06); 2) attitudes (questions 07 to 11), and 3) practices (questions 12 to 19). Another instrument was developed for the sociodemographic characterization of the target population, consisting of caregivers from LTCFs in the city of João Pessoa, state of Paraíba.

The instrument content was assessed by the experts using the Delphi technique in two rounds (Delphi I and II). For the semantic analysis of the instrument items, they were read individually by the students of the caregiver course, who evaluated the understanding of the words and suggested changes in the sentences^(10)^. After that, an appearance analysis was conducted by the higher education professors, as part of the semantic analysis^(10,13-14)^, with suggestions for readjustment of some terms.

After the process of semantic and appearance analysis, sociodemographic variables were added, giving the final version of the instrument 25 KAP items addressing PI prevention, with 7 questions related to knowledge (1 to 7), 8 questions related to attitudes (8 to 15), and 10 about practices (16 to 25).

### Study and statistical analysis

**Figure 1:**
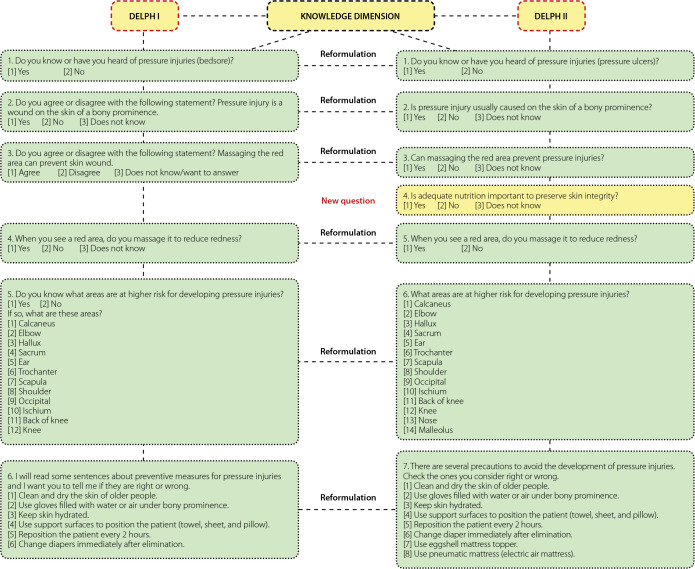
Instrument changes in the knowledge dimension during the stage of content validation by the experts, Delphi I and II rounds, João Pessoa, Paraíba, Brazil, 2019

For the analysis of the items comprising the instrument, the acceptance criterion was defined as ≥0.80 for the content validity index (CVI), more specifically by the I-CVI, which measures the proportion of experts who are in agreement regarding the items of the instrument^(14)^. I-CVI values <0.80 determined the reformulation and/or exclusion of each item^(15-16)^, as indicated in the results (Figure 1).

Descriptive statistics were used to characterize the study participants, with calculation of absolute and relative frequency for the categorical variables, and mean and standard deviation for the variables of age and professional experience. The results are presented in tables and concept maps (CmapTools 6.01).

Reliability of the experts’ agreement of the assessment was also analyzed using Kappa coefficient. For Landis and Koch (1977), the K measure suggests the following interpretation: <0 - no agreement; 0 to 0.19 - poor, 0.20 to 0.39 - reasonable, 0.40 to 0.59 - moderate, 0.60 to 0.79 - substantial, 0.80 to 1.00 - excellent/almost perfect.

## RESULTS

### Instrument content analysis (n=10)

Professionals with master’s and doctor’s degrees in nursing participated in the instrument content assessment. Most of them were female, with a mean age of 46.80 (SD±11.361), and a mean professional experience of 23.5 years (SD±11.326). According to an adapted version of the Fehring model^(12)^, two participants obtained 9 points, two 10 points, two 11, and four 14 points, all above the minimum score recommended by the model.

The assessment scores of all 19 items provided by the experts in the Delphi I round were obtained through the I-CVI, and the items with an agreement equal to or higher than 80% remained in the instrument. For the analysis of inter-observer reliability, the Kappa coefficient in the first round was >0.76, showing a median/substantial level of agreement (Table 1).

**Table 1:** Item Content Validity Index in Delphi I round, according to the criteria of clarity and relevance, João Pessoa, Paraíba, Brazil, 2019

Variables	Delphi I	
	Clarity	Relevance
	I-CVI^*^	I-CVI^*^
Questions addressing Knowledge		
1. Do you know or have you heard of pressure injuries (bedsore)?	0.70^**^	0.90
2. Do you agree or disagree with the following statement? Pressure injury is a wound on the skin of a bony prominence.	0.60^**^	1.00
3. Do you agree or disagree with the following statement? Massaging the red area can prevent skin wound.	0.30^**^	0.70^**^
4. When you see a red area, do you massage it to reduce redness?	0.60^**^	0.70^**^
5. Do you know what areas are at higher risk for developing pressure injuries?	0.60^**^	0.90
6. I will read some sentences about preventive measures for pressure injuries and I want you to tell me if they are right or wrong.	0.70^**^	0.80
Questions addressing Attitudes		
7. Do caregivers have a critical role in maintaining the skin integrity of institutionalized older people?	0.80^**^	1.00
8. Is it important to encourage repositioning of bedridden older people?	0.80^**^	0.80
9. Should caregivers observe eating problems in older people?	0.60^**^	0.90
10. Are caregivers essential in the care process of institutionalized older people?	0.70^***^	0.60^***^
11. Is it important to massage the red bony prominences?	0.60^***^	0.70^***^
Questions addressing Practices		
12. Do you reposition bedridden older people?	0.70^**^	1.00
13. Regarding skin care of older people:	0.40^**^	0.90
14. Do you check the skin watching for changes?	0.90	1.00
15. Do you try to keep bed sheets tight?	0.60^**^	0.60^**^
16. Do you use any material to support any area of the body?	0.60^**^	0.90
17. Regarding the materials, which one(s) do you use to support the body area?	0.60^**^	0.90
18. Do you check if the older people accept the diet well?	0.80	0.90
19. Do you offer liquids (water/juice) to older people?	0.80	0.90
Scale content validity index	0.66	0.85
Kappa coefficient of agreement	0.76	

* I-CVI: Item content validity index;

** Reformulated items;

*** Excluded items.

**Figure 2:**
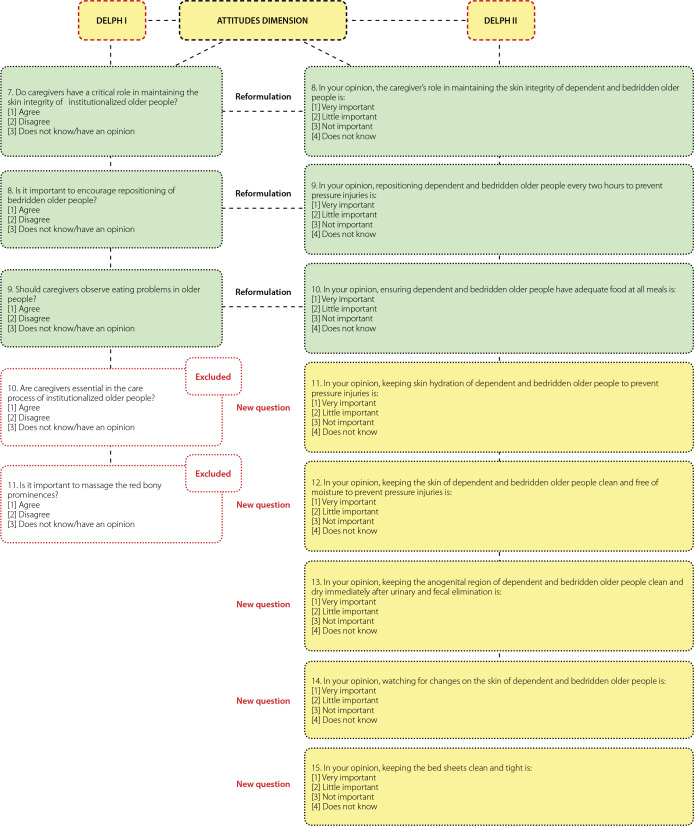
Instrument changes in the attitudes dimension during the stage of content validation by the experts, Delphi I and II rounds, João Pessoa, Paraíba, Brazil, 2019

**Figure 3:**
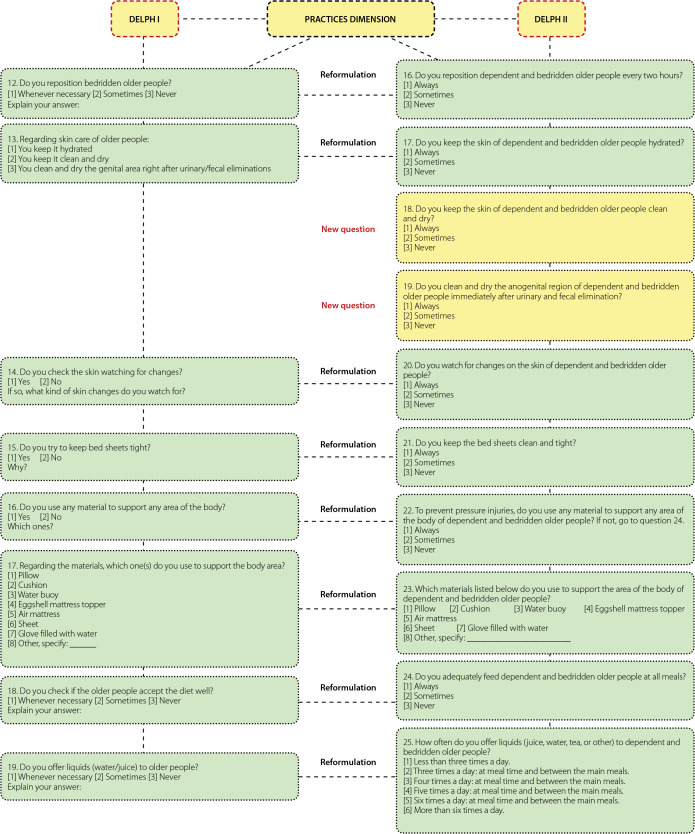
Instrument changes in the practices dimension during the stage of content validation by the experts, Delphi I and II rounds, João Pessoa, Paraíba, Brazil, 2019

Figures 1, 2, and 3 show the changes recommended by the participants in the items that obtained agreement indices below 0.80.

The I-CVIs in the Delphi II round were higher in 19 of the 25 instrument items for the criteria of clarity and relevance. The S-CVI for the criterion of clarity was 0.95 and for relevance, 1.00. For the analysis of inter-observer reliability, the Kappa coefficient in the second round was >0.97, which indicates excellent agreement (Table 2).

**Table 2:** Item Content Validity Index in Delphi II round, according to the criteria of clarity and relevance, João Pessoa, Paraíba, Brazil, 2019

Variables	Delphi I	
	Clarity	Relevance
	I-CVI^*^	I-CVI^*^
Questions addressing Knowledge		
1. Do you know or have you heard of pressure injuries (pressure ulcers)?	1.00	1.00
2. Is pressure injury usually caused on the skin of a bony prominence?	0.80	1.00
3. Can massaging the red area prevent pressure injuries?	1.00	1.00
4. Is adequate nutrition important to preserve skin integrity?	0.90	1.00
5. When you see a red area, do you massage it to reduce redness?	0.80	1.00
6. What areas are at higher risk for developing pressure injuries?	1.00	1.00
7. There are several precautions to avoid the development of pressure injuries. Check the ones you consider right or wrong.	1.00	1.00
Questions addressing Attitudes		
8. In your opinion, the caregiver’s role in maintaining the skin integrity of dependent and bedridden older people is:	1.00	1.00
9. In your opinion, repositioning dependent and bedridden older people every two hours to prevent pressure injuries is:	1.00	1.00
10. In your opinion, ensuring dependent and bedridden older people have adequate food at all meals is:	1.00	1.00
11. In your opinion, keeping skin hydration of dependent and bedridden older people to prevent pressure injuries is:	0.90	1.00
12. In your opinion, keeping the skin of dependent and bedridden older people clean and free of moisture to prevent pressure injuries is:	1.00	1.00
13. In your opinion, keeping the anogenital region of dependent and bedridden older people clean and dry immediately after urinary and fecal elimination is:	1.00	1.00
14. In your opinion, watching for changes on the skin of dependent and bedridden older people is:	1.00	1.00
15. In your opinion, keeping the bed sheets clean and tight is:	1.00	1.00
Questions addressing Practices		
16. Do you reposition dependent and bedridden older people every two hours?	0.90	1.00
17. Do you keep the skin of dependent and bedridden older people hydrated?	1.00	1.00
18. Do you keep the skin of dependent and bedridden older people clean and dry?	1.00	1.00
19. Do you clean and dry the anogenital region of dependent and bedridden older people immediately after urinary and fecal elimination?	1.00	1.00
20. Do you watch for changes on the skin of dependent and bedridden older people?	1.00	1.00
21. Do you keep the bed sheets clean and tight?	1.00	1.00
22. To prevent pressure injuries, do you use any material to support any area of the body of dependent and bedridden older people? If not, go to question 24.	0.90	1.00
23. Which materials listed below do you use to support the area of the body of dependent and bedridden older people?	1.00	1.00
24. Do you adequately feed dependent and bedridden older people at all meals?	1.00	1.00
25. How often do you offer liquids (juice, water, tea, or other) to dependent and bedridden older people?	1.00	1.00
Scale content validity index	0.95	1.00
Kappa coefficient of agreement	0.97	

* *I-CVI: Item content validity index.*

### Semantic analysis (N=45)

After content validation by the experts, the semantic analysis of the 25 items was performed with students in the last year of a technical course for caregivers of older people at the Health Technical School, which belongs to the federal education network. In this stage, suggestions were provided to improve the understanding of items 11 and 25.

Regarding the statement of item 11, “*To prevent pressure injuries, do you think that keeping the skin of dependent and bedridden older people hydrated is*”, an adjustment was suggested to: “*To prevent pressure injuries, do you think that keeping the skin hydration of dependent and bedridden older people is”.* In item 25, the suggested alteration was to the answer, from *“six or more times a day”* to *“six times a day, at the time and between the main meals”,* and inclusion of another answer option: *“more than six times”.*

### Appearance analysis (N=23)

After the semantic analysis, the instrument was submitted to an appearance validation by 23 nursing professors who suggested changes only in item 25. The reformulated question would make its construction more adequate without affecting the suggestions in the semantic analysis. Therefore, the addition of the following phrase was proposed: ... *at meal time and between the main meals* in the answer option *four times a day*.

## DISCUSSION

The construction and validation of the instrument to assess the knowledge, attitudes, and practices (KAP) of caregivers related to preventive measures for pressure injuries in older people living in long-term care facilities is an important technological product that can be used in institutions by nurses to collect information, and identify weaknesses and strengths of caregivers regarding PI prevention, allowing proposals of educational interventions specifically focused on eliminating weaknesses and reinforcing proper constructs of knowledge, attitudes, and practices for the provision of more assertive care by caregivers.

The development and validation of this educational content validation instrument in health contribute to clinical and scientific practices, as it is an innovative tool to validate educational content available in materials such as videos, albums, booklets, games, websites, and software applications, supporting health education activities as it does not specify information regarding the topic, target audience, and circumstances of use^(16)^.

For the instrument development and validation, recommended operational stages were observed with scientific rigor in order to build high-quality, clear and relevant items, indicating the legitimacy and credibility of the results of studies where it will be used, which reinforces the importance of the validation process and the quality of the attributes to be achieved^(17-18)^.

Experts were selected using the Fehring model, which has very specific parameters for the minimum and maximum scores (5 and 14 points, respectively). This model was adapted to our study and presented 11 and 14 points as the minimum and maximum scores, respectively. Thus, the higher the score obtained, the higher the reliability of the assessment and content validation^(12)^.

Regarding their profile, the sample had only female experts, with a mean age of 46.8 years, and mean professional experience of 23.5 years. Nursing is closely associated with typically female care, and when studying the history of nursing and the contributions of Florence Nightingale, the care description clearly assigns the task to women^(19)^, a characteristic that is still seen in the profession today. Also important, professional experience is essential for the instrument assessment, since there is a more consolidated judgment in the processes of professional training and care provision.

Delphi I round showed 14 items (1, 2, 3, 4, 5, 6, 9, 10, 11, 12, 13, 15, 16, 17) for reformulation due to clarity, and five (3, 4, 10, 11, 15) due to relevance, with I-CVI <0.80. Although items 7 and 8 had reached I-CVI ≥0.80, they received suggested reformulations. For this reason, items 10 and 11 of the attitudes construct were excluded from the instrument, as they presented poor clarity and relevance. The scores indicated necessary changes in the items inherent to the knowledge, attitudes, and practices constructs (Table 1).

Considering the results for the Delphi I round, the I-CVI obtained in the clarity criterion was ≤0.66 and in the relevance criterion ≥0.85. However, regardless of a good assessment of relevance, the items of the constructs considered favorable for change by the experts had to be reformulated. Of note, the suggestions provided by the experts were important to make the questions clearer and more relevant to the target population and the topic addressed.

The Kappa coefficient of agreement, by Landis and Koch^(20)^, was used for the reliability analysis. This is an appropriate statistical procedure to measure the reliability of general agreement items. In the first round, the Kappa value was >0.76, that is, median/substantial level of agreement, and in the sequence the instrument was restructured.

In the Delphi II round, the I-CVI for clarity was 0.95 and relevance 1.00, and the S-CVI was 0.97. Reformulated and excluded items ensured adequate content and structure of the instrument, according to the analysis of the experts. For the reliability analysis, the Kappa value was >0.97, indicating excellent agreement among the raters.

Regarding the suggested changes in the items of the instrument, a new item was added, question 4, and knowledge-related items 1, 2, 3, 5, 6, and 7 were reformulated. In the attitudes section, three items were reformulated (8, 9, and 10), and five were added (11, 12, 13, 14, 15). For questions addressing practices, 8 items were reformulated (16, 17, 20, 21, 22, 23, 24, and 25), and two new items were added (18 and 19) (Figures 1, 2, and 3).

After the Delphi II round, the agreement scores were improved for clarity and relevance, as seen in the I-CVI and S-CVI of the instrument, and the higher Kappa agreement coefficient.

After completing the Delphi I and Delphi II rounds, the semantic analysis was performed, which allowed participants to read the instrument and indicate words and/or sentences that made the instrument more difficult to understand. In this stage, of the 25 items organized in the instrument, changes were suggested in one item related to attitudes (item 11) and one item related to practices (item 25). This phase is very important, since a poor understanding of a question tends to impact the instrument applicability to the target audience^(21)^.

After the semantic analysis, an appearance analysis was performed by nursing professors, who analyzed the suggested changes in the items. In this stage, the participants had to evaluate the changes so that the instrument did not contain colloquial words that could make it inelegant^(10,14,22)^. The changes made were only in item 25, related to practices.

Validation is a decisive factor in the selection and/or application of a measurement instrument and can be assessed through the concept the instrument proposes to measure^(22)^. Therefore, validation studies are important as they determine the legitimacy and credibility of the results of a study and the recognition of the instrument quality^(15)^.

Instrument validation studies in health must be conducted with attention to methodological rigor, so that the instrument content can provide satisfactory contributions through assessments and/or reformulations suggested by experts in order to improve and qualify the tool^(12)^.

It should be noted that validated instruments with a focus on the knowledge, attitudes, and practices of caregivers of institutionalized older people about the prevention of pressure injuries can promote changes in this population’s reality and more effective intervention strategies that help reduce collective problems leading to PI development in older people and, consequently, promote better quality of life. In addition, health behaviors should be aligned with scientific knowledge, which in turn, can favor the adoption of adequate attitudes and, consequently, good health practices^(2,23)^.

Instrument construction based on the KAP survey shows a perspective of health behavior linked with the acquisition of scientific knowledge that can lead to favorable attitudes and good health practices, based on the idea that such behavior is connected with the values and beliefs of people^(23)^. In this sense, the instrument constructed in our study promotes a new look and innovation with the potential to identify weaknesses of caregivers of older people in one or more studied constructs, highlighting relevant aspects for future actions of PI prevention in older people with restricted mobility living in long-term care facilities.

### Study limitations

A representative sample of the target population is recommended for semantic validation. In this study, students from a technical caregiver course, in the conclusion phase, were selected as the sample not to compromise the sample size in subsequent studies, observing as much as possible the steps of the proposed method to obtain relevant contributions to instrument understanding. Thus, the strategy adopted in this study was considered successful.

### Contributions to nursing

The contribution of this study consists in providing an assessment of the knowledge, attitudes, and practices of caregivers of older people regarding the prevention of pressure injuries in older people. The results of this assessment will enable the development of educational strategies to fulfill the needs of the target audience, potentially engaging the participants due to the perceived affinity of ‘findings versus educational proposal’ with their professional responsibilities in daily care, to promote well-being and quality of life for dependent and/or bedridden older people living in long-term care facilities.

## CONCLUSIONS

Our study produced a valid measurement instrument in terms of content and structure to assess the knowledge, attitudes, and practices of caregivers of institutionalized older people regarding PI prevention. It uses formal language and contains sentences and words that are easily understood by the target audience in order to support nurses who organize and manage activities performed by caregivers of older people in the identification of weaknesses that may directly affect the provision of care and maintain the skin integrity of older people.

The assessment by the experts - students of a technical caregiver course and nursing professors - led to reformulations of items in constructs to obtain proper scores in the assessment of content validity index and Kappa coefficient values, reaching an excellent level of agreement.

Further studies, with a pilot test and assessment of the psychometric properties of the instrument “Inquiry Knowledge, Attitudes, and Practices Survey with Caregivers of Older People on Pressure Injury Prevention” will allow additional reliability analysis while measuring the proposed constructs - knowledge, attitudes, and practices regarding the object of interest - improving the confidence in the instrument and ensuring a lower risk of measurement errors, finally providing researchers, managers, and health professionals with a tool to help improve the state of the art and the quality of care in the prevention of pressure injuries in dependent and bedridden older people.
